# Sustainable Development of Innovative Green Construction Materials: A Study for Economical Eco-Friendly Recycled Aggregate Based Geopolymer Concrete

**DOI:** 10.3390/ma13214881

**Published:** 2020-10-30

**Authors:** Hatem Alhazmi, Syyed Adnan Raheel Shah, Atif Mahmood

**Affiliations:** 1 National Center for Environmental Technology (NCET), Life Science and Environment Research Institute (LSERI), King Abdulaziz City for Science and Technology (KACST), P.O. Box 6086, 11442 Riyadh, Saudi Arabia; halhazmi@kacst.edu.sa; 2 Department of Civil Engineering, Pakistan Institute of Engineering and Technology, Multan 66000, Pakistan; 3 Instituut Voor Mobiliteit, Universiteit Hasselt, Wetenschapspark 5 bus 6, 3590 Diepenbeek, Belgium; 4 Department of Industrial and System Science Engineering, Binghamton University State University of New York, New York, NY 13902, USA; Amahmoo6@binghamton.edu

**Keywords:** sustainability, geopolymer concrete, recycled aggregate concrete, eco-friendly

## Abstract

Green revolution and high carbon footprint concepts have attracted the development of a green and sustainable environment. This work endeavors to investigate the behavior of recycled aggregate geopolymer concrete (RAGC) developed with four different types of effluents to develop sustainability in the construction industry and to produce an eco-friendly environment. Each of the types of effluents was used by completely replacing the freshwater in RAGC to examine its influence on compressive strength (CS), chloride ion migration (CIM), split tensile strength (STS), and resistance to the sulfuric acid attack of RAGC at various testing ages. The test outputs portray that the effluent obtained from the textile mill performed well for the CS (25% higher than the control mix) and STS (17% higher than the control mix) of RAGC. Similarly, the highest mass loss of RAGC due to the acid attack (41% higher than control mix) and the highest CIM (29% higher than control mix) were represented by the RAGC mix made with effluent obtained from fertilizer mill. The statistical analysis indicated no significant influence of using textile mill effluent (TE), fertilizer mill effluent (FE), and sugar mill effluent (SE) on the STS, CIM, and mass loss due to acid attack while it presented a significant influence on the CS of various mixes. Therefore, this investigation solidly substantiates the acceptability of studied types of effluents for the fabrication of eco-friendly green materials.

## 1. Introduction

For the development of green materials, use of recycled aggregate concrete (RAC) resulting from the demolition of existing infrastructure is growing which is influenced by the global population, rapid urbanization, and economic scenario of developing countries. Hence, this construction and demolition waste must be used properly to promote a sustainable ecosystem. The RAC reduces carbon footprint, aggregates transport distances, and disposal space available for building and demolition waste by satisfying the demand for environmentally safe, low carbon and green construction [1,2,3,4,5]. While RAC has many inadequacies compared to natural aggregate concrete (NAC), such as low split tensile strength (CS), high water absorption (WA), and high porosity but RAC significantly improves ductility which is still one of the most conservative benefits [6]. CO_2_ emission is a by-product obtained during Portland cement manufacturing. In the present research, a green concrete called “geopolymer concrete” (GPC) made of recycled coarse aggregates (RCA) was utilized to reduce the carbon footprint of concrete construction. GPC concrete uses a binder made of inorganic alumino-silicate polymers formed by alkaline activators like silica fumes, fly ash, blast furnace slag, and red mud. Besides, many urban run-offs and industrial effluents are discharged into rivers and landfills. Besides this, due to strict criteria by environmental regulatory authorities and denial of open dumps near communities, the pressure is building to seek alternative means of dumping at a sufficient expense. The rapid inhabitant expansion and the rise in economic activity have spurred freshwater supply. Concrete, the second most widely used material after wood, which consumes one trillion gallons of water every year [7]. Hence, to establish a balance between requirement and freshwater supply, the use of freshwater must be brought down, particularly in the construction industry or other sectors [8]. According to the study, 50% of the world population will be drained of freshwater by 2020 [9]. People are growing importance of the recycle of effluent, especially in concrete. The high cost of treating effluent may be minimized instead of using it to make concrete [10]. Additionally, this contaminating water seriously affects the natural environment and human health. Thus, these detrimental effects on the climate and living being biodiversity could be tackled to some extent using effluent in concrete manufacturing.

To date, several studies have examined the fresh, mechanical, and durability properties of RAC produced by freshwater and concluded that the RAC has shown superior qualities compared to typical concrete [5,11,12,13]. Some research suggests that the CS is reduced between 10–20% by increasing the substitution of natural coarse aggregates (NCA) with RCA [14,15,16]. The CS of RAC was reduced by 20–25% compared to traditional concrete by maintaining the cement content and water-to-cement ratio stable, when NCA was replaced by RCA [17]. To ensure a better CS of RAC it is important to procure the RCA from a single source. If the RCA is derived from different resources, the difference in the CS could become more prominent due to the disparity of aggregate properties [17,18,19,20,21]. The mortar which adheres to coarse aggregates also influences the strength properties of RAC. It was noted that the CS of RAC decreased by up to 10% corresponding to 34% adhered mortar for a maximum size of 10–20 mm [21]. The literature has highlighted many studies showing the use of various forms of effluent in the manufacture of concrete and other construction materials. A detailed analysis of alkali-activated concrete containing recycled wash water and found that recycled wash water did not have any harmful effects on alkali-activated concrete development [22]. Furthermore, the strength of the mortar cubes casting from the water of waste treatment plants did not show significant differences from freshwater [23]. Detailed analysis of the compressive behavior of concrete cast from treated effluent found that the CS increased up to 28 days by increasing the volume of treated effluent [24]. Besides, it was observed that when treated effluent used for curing then CS increased up to 1.5%. The strength of concrete increases by 9% when tap water is used instead of freshwater and when treated domestic sewage takes over freshwater there is no impact on the setting time [25]. The concrete produced with wash water gave CS of 96% in comparison to that produced with freshwater [26]. The setting time and the strength properties of concrete improved while using treated effluent rather than freshwater [27,28]. It was observed that the CS of concrete was improved by 17% after 180 days of testing while using effluent after primary and secondary treatment of effluent. Later, axial strength has shown a declining strength by 18% in the case of secondary treated effluent, separately. The concrete produced with secondary treated effluent also showed a higher WA value [29]. The results of concrete developed employing concrete wash water revealed that a good quality of fresh concrete could be produced using concrete wash water [30]. Results demonstrated that concrete built with concrete wash water or a mixture of effluent and freshwater was shown inferior CS properties as compared to concrete developed using silica and additive admixture water [31]. Wasserman [32] concluded that concrete manufacturing using concrete wash-water offered better strength compared to freshwater after studying the comparative analysis of results. Nikhil et al. [33] studied the CS of concrete produced with three distinct types of effluent such as sewage water, groundwater and freshwater and reported that concrete produced using freshwater gave higher CS. Rabie et al. [34] findings on the automated properties of concrete created by adding different forms of effluent sludge deduced that by adding a small amount of sludge (5%, 10%, and 15% by weight of cement), the impact on the CS of concrete was minimal, but after replacing cement by 20% with dry and wet sludge, concrete compresses were reduced by 61.6% and 68.5%. Roychand et al. [35] studied the impacts on concrete mechanical properties of steel slag collected from waste treatment plants and reported that by replacing CA with SSA, the CS of the concrete raised by 18–16.8% at 7–28 days, separately. Tembhurkar [36] examined bio-concrete activity by incorporating effluent and steel slag and concluded that the decline in bio-concrete properties could be resolved through the use of microbiologically induced calcium carbonate. Tensile and CS was increased by 12.5% and 31.1% separately with decreased WA of bio concrete. Due to the difference in water’s pH value, mass loss of concrete is a serious issue. The reduction in the pH of the mixing water shows an increase in concrete disintegration [37,38].

This study focuses on the use of RCA and effluent in geopolymer concrete could help to create a sustainable environment. Hence, a detailed and comprehensive study is essential to explore the physical and durability properties of recycled aggregate geopolymer concrete (RAGC) produced by various types of effluent. This study aims to investigate the mechanical properties (CS and split tensile strength (STS)) and durability properties (chloride ion migration (CIM) and sulfuric acid attack resistance) of RAGC mixes at different curing ages by utilizing different forms of effluent, such as sugar mill effluent (SE), fertilizer mill effluent (FE), textile mill effluent (TE). A control RAGC mix made with freshwater was fabricated for the comparison purpose. Additionally, the significance of the variation between different properties of RAGC mixes was observed by one-way analysis of variance test. In this study, a sustainable and green development approach is recommended by limiting the use of new natural resources with the concept of circular economy.

## 2. Materials and Methods

### 2.1. Materials

**Figure 1:**
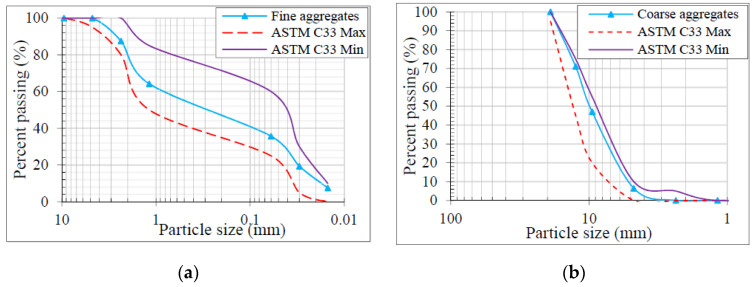
Granular analysis (**a**) fine aggregates (**b**) RCA.

The coarse aggregates were replaced with RCA during the preparation of GRAGC. The RCA has been extracted from crushing concrete cylinders and having the CS varying from 30 MPa to 45 MPa at 6 months to 1 year of age. Different properties of recycled aggregates have been shown in Table 1 according to ASTM C33 [39]. The maximum size of the recycled aggregate was 10 mm. The Lawrancepur sand was used which is locally available and having an apparent particle density of 2586 kg/m^3^ with a fineness modulus of 2.25. The granular analysis of RCA and sand used in the current study is shown in Figure 1. In this study, class F fly ash (60%) and soil granulated blast furnace slag (GGBS, 40%) were used as binders in GRAGC which is locally available in the market. Various features of fly ash were reported in Table 2. In the mass ratio of 1:2.5, a combination of NaOH (14 M molarity) and Na2SiO3 was employed as an activator. For fresh testing, a slump test was performed as per ASTM C143/C143M-15 [40] and reported a slump value of 125 mm in the case of fresh GRAGC. According to ASTM C807-13 [41], a setting time of 90 min was recorded. To verify the CS of GRAGC, six 150 mm × 300 mm concrete cylinders were filled during the casting of column specimens. When the columns were measured, the cylinders were also measured on the same day. The GRAGC showed an average strength of 34.5 MPa with a mean difference of 2.27 MPa.

Four different forms of effluent collected from their sources have been used for RAGC production. Each of the forms of effluent was fully replaced with freshwater. The chemical properties of all effluent forms used in the present work are described in Table 3. The effluent samples their chemical properties.

### 2.2. Manufacture and Testing

Four RAGC mixture were produced using various forms of effluent called freshwater mix (FW), sugar mill effluent mix (SF), fertilizer mill effluent mix (FF), textile mill effluent mix (TF). The RAGC mix prepared with freshwater (FW) was evaluated and then their results are compared with other groups developed using different types of effluent. The quantity of any form of effluent used was equivalent in all these RAGC mixes. From each of these RAGC blends, a total of eighteen (18) separate cylindrical specimens having a measurement of 150 mm in diameter and 300 mm in height were produced to find the effects of compressive as well as STS of the three diverse samples at all curing ages. The specimens made to examine the migration of chloride ions were thirty-six (36) (having a height of 100 mm and a diameter of 100 mm). There were fifty-four (54) specimens made to investigate the resistance against sulphuric acid attack (cubes having 100 mm side length). After trial testing, a mixed design of GPC having a nominal density of 2400 kg/m^3^ was prepared as shown in Table 4 and WA of RCA was also considered.

By using a mechanical mixer with a volumetric capacity of 0.15 m^3^ and a speed of 20 revolutions/min, the concrete was mixed, and the mixing time was 10 min. The aggregates were combined in the first half of the period with half of the water accompanied by adding remaining water as well as cement then mixing was done for 5 min to ensure a homogenous RAGC mix. A slump test was conducted according to ASTM C143 [44] which gave slump values varying between 85 mm to 110 mm for all the types of effluent. For the curing of all the RAGC mixes, ordinary water was used. Compressive and STS were two main mechanical properties found for each RAGC mix at different ages. The CS was found at 7, 28, and 90 days as per [45] whereas STS was tested at the same ages according to ASTM C496 [45]. The durability characteristics such as chloride ion penetration and acid attack were investigated for every four different forms of the RAGC mixes. 

To assess the resistance of the specimen against sulphuric acid, the curing of specimens in normal temperature water for 28 days allowed to dry at 50 °C for 24 h and then dipping them in 4% H_2_SO_4_ for an acid attack. The samples were tested for their mass loss after being submerged for 28, 90, and 120 days. Similarly, for the determination of chloride ion penetration, the prepared samples were first treated in ordinary water for 28 and 90 days, then oven-dried for 24 h at a temperature of 50 °C. This was accompanied by the samples being cooled to room temperature and then immersed in a solution of 4% NaCl for 56 days. This was followed by the step involved in the splitting of cylinders as per ASTM C496 [45] then dispersed in water with 1 N solution of AgNO_3_. To determine the migration of chloride ions for concrete formed by each type of effluent, the chemical reaction of AgNO_3_ with the chloride ions that have penetrated, makes AgCl showing off the silver color, was carried out. 

## 3. Results and Analysis

### 3.1. Compressive Strength (CS)

**Figure 2:**
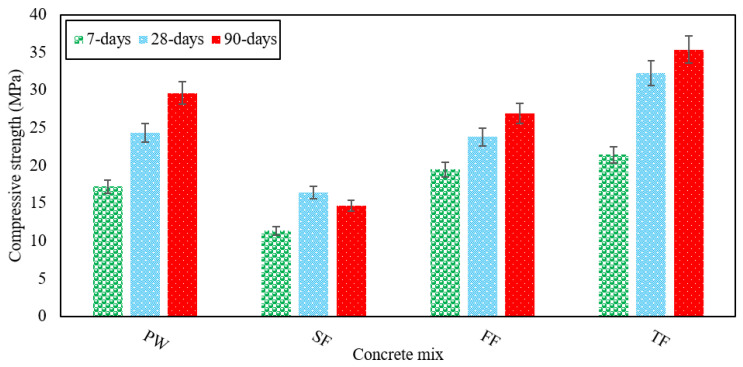
CS of various RAGC mixes at 7, 28, and 90 days of testing.

In this study, CS was observed for each of the four separate RAGC mixes at 7, 28, and 90 days of testing following ASTM C39 [46]. Figure 2 shows the CS of each varying RAGC mix. For each age group, three produced samples of all RAGC mixes were put in a compression testing machine, and then their mean results were determined. The maximum CS was shown by the TF mix, while the lowest CS was shown by the FF RAGC blend at all the various test ages. The control mix (FW) was produced to carry out a detailed analysis of the results of various other RAGC mixes made using various forms of effluent. After 7 days of testing, the CS of 19 MPa was recorded in the case of FW, and CS improved by 21% have a value of 24 MPa after 28 days of testing. The CS was 27 MPa when measured at 90 days, which was 130% of the CS calculated at 7 days. Hence, the FW mix has shown continuous improvement in its CS with the curing ages.

In contrast to FW at all ages, the concrete CS improved substantially for the TF mix. The TF mix had shown a CS of 22 MPa at 7 days, which was 20% higher than the FW mix at 7 days. The TF mix demonstrated a significant improvement of 133% in CS at 28 days with a value of 32 MPa that was on average 25% higher than the FW mix. At 90 days of testing, the FF mix reported a higher strength of 35 MPa but it was on average 16% higher than the strength shown for FW. When using TE to produce RAGC mixes, the CS observed was higher compared with the FW mix. The fluoride and bicarbonates contained in TE experience a reaction with Al_2_O_3_ found in OPC and fly ash leading to calcium fluoro aluminate structure resulting in better CS. This mineral is highly reactive that results in fast-setting as well as early hydration of the cement thus increasing the strength [36]. The fly ash proved to be effective for the CS of RAGC due to the pozzolanic reaction between CH and fly ash particles. The adding of fly ash to concrete advances the particle size distribution in the concrete matrix by filling the voids between cement and sand particles and changes the binder mix to CSH-gel giving a strong bond.

It was noted that when SE was used for RAGC mix, CS was higher at 7 days and lower at 28 and 90 days compared to FW. After 7-day testing, the SF mix indicated CS of 11 MPa which was on average 33% lower than the FW strength measured at 7 days. At 28 days, the SF mix reported an increase of 31% with a value of 16 MPa in CS. The FF mix showed a decline of 11% in its CS at 90 days relative to that at 28-days, but this CS was 50% lower than the CS of FW at 7-days. Due to the rising amount of BOD and COD in the FE resulting in a reduction in the strength of the FF mix at the 90-day testing [47]. This reduction in strength can be due to the SE components. As SE was combined, it was noted that the water to cement ratio had increased, reducing the strength of the FF mix. The concrete CS is also substantially impacted by the presence of organic mixed waste. 

**Figure 3:**
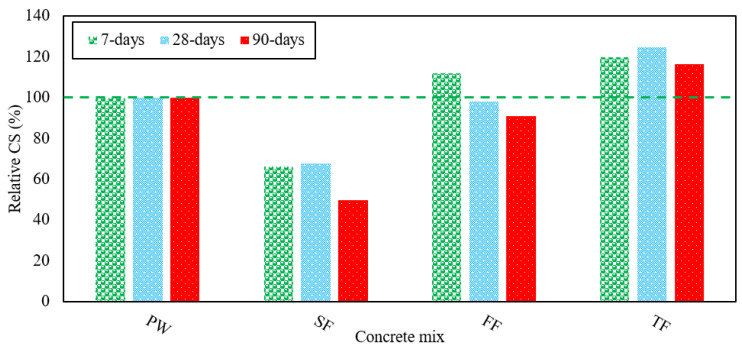
CS of various RAGC mixes relative to FW at 7, 28, and 90 days of testing.

These wastes tend to accumulate water during the mixing process and later release this water when they are condensed in concrete while raising the water-cement ratio, and is one of the key reasons for the reduction in CS, can also be due to the presence of organic impurities, such as a large amount of sulfate in SE. The large number of organic compounds in SE that react with cement components and thus inevitably affect the concrete strength and resulting in this decrease in strength of the SF mix. Due to the large quantities of sulfate found in SE, the CS of the SF mix is reduced after 90 days of testing. The relative CS of various RAGC mixes was shown in Figure 3. Thus, the formation of CSH gel in RAGC enhances the CS of all mixes.

### 3.2. Split Tensile Strength (STS)

**Figure 4:**
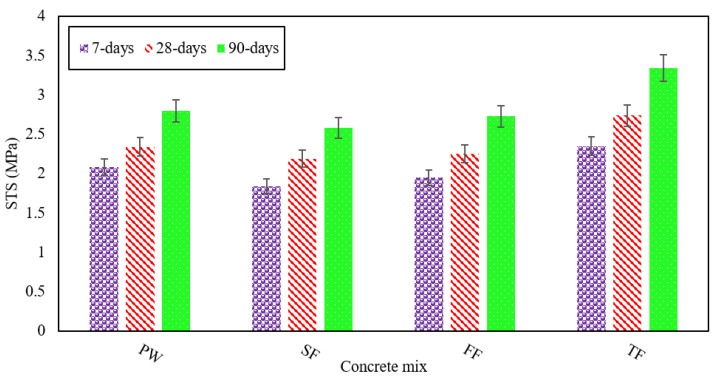
STS of various RAGC mixes at 7, 28, and 90 days of testing.

Figure 4 illustrates the STS as shown by various RAGC mixes which were produced using different effluent types. These experiments were conducted at 7, 28, and 90 days of casting according to ASTM C496 [45]. The average STS of the FW mix was 2 MPa at 7 days, 2.3 MPa at 28 days, and 2.8 MPa at 90 days, separately. This indicates that an STS of 117% of its strength at 28 days was shown by the FW mix at 90 days. The highest STS was shown by the TE mix, while the lowest was shown by the FF mix.

The TF mix displayed improved STS of 2.3 MPa at 7 days, 2.7 MPa at 28 days, and 3.3 MPa at 90 days, and was on average 11%, 14%, and 16% higher than those of the FW mix measured at 7, 28, and 90 days, separately. The tensile strength demonstrated by the TF mix was better, the reason is that TE has lower bicarbonates than the other forms of effluent. It seems that the rise in bicarbonates contributes to a decrease in concrete tensile strength [48]. The fly ash augmented the STS of RAGC mixes due to development in the particle size distribution in the concrete matrix by filling the voids between cement and sand particles. It changes the binder mix to CSH-gel giving a strong bond between the particles of RAGC.

**Figure 5:**
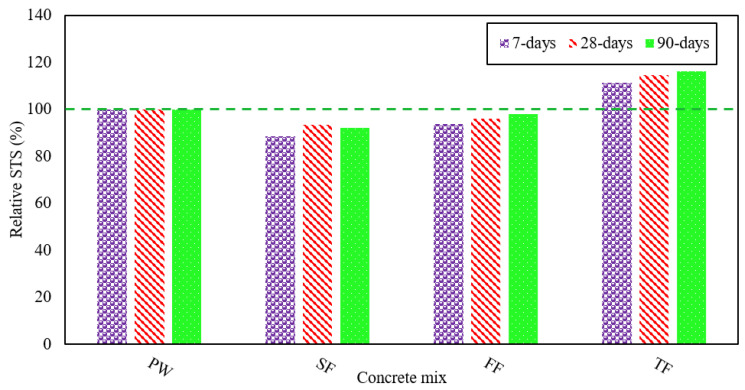
STS of various RAGC mixes relative to FW at 7, 28, and 90 days of testing.

The FF mix displayed STS of 1.9 MPa at 7 days, 2.2 MPa at 28 days, and 2.7 MPa at 90 days. This suggests that the STS were reduced by 6% at 7 days, 4% at 28 days, and 2% after 90 days of testing when FE was used for mixing. For each type of effluent decline in STS was studied and analyzed. In contrast to the FW mix, the tensile strengths indicated by the SF mix were lower such as 11% at 7 days, 6% at 28 days, and 7% at 90 days. The SF mix displayed STS which were on average of 1.8 MPa at 7 days, 2.2 MPa at 28 days, and 2.6 MPa at 90 days. The decrements in STS shown for various RAGC mixes (i.e., FF and SF) could be related to the reasons that BOD, COD, as well as total suspended solids (TSS), are rich in such types of effluent [49]. When the level of chloride is expanded, the concrete exhibited a reduction in its tensile strength [50], this in turn induced a decrease in the tensile strengths displayed by the chloride-rich RAGC mixtures. These blends also had a lower pH-value. The reduction in STS is due to a decrease in pH value [51]. Figure 5 shows the contrast between the average % age of STS displayed by various RAGC mixes and that shown by the FW mix at different ages.

### 3.3. Relationship between CS and STS

**Figure 6:**
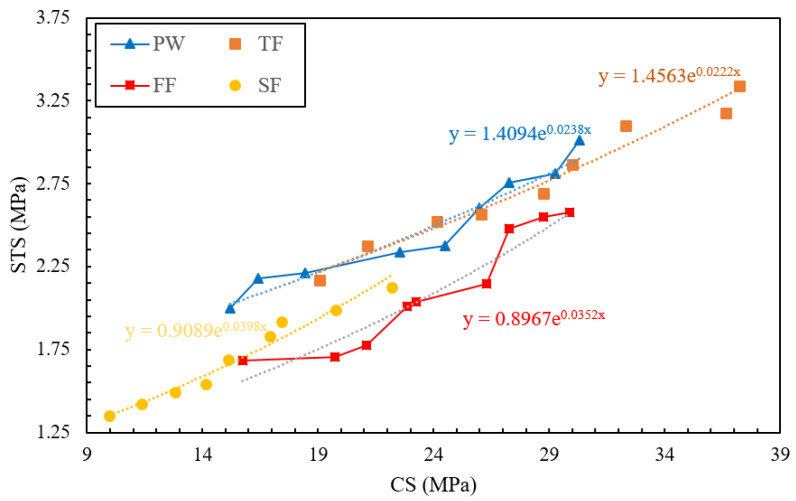
Predictions of various codes for the CS and STS of RAGC mixes.

**Figure 7:**
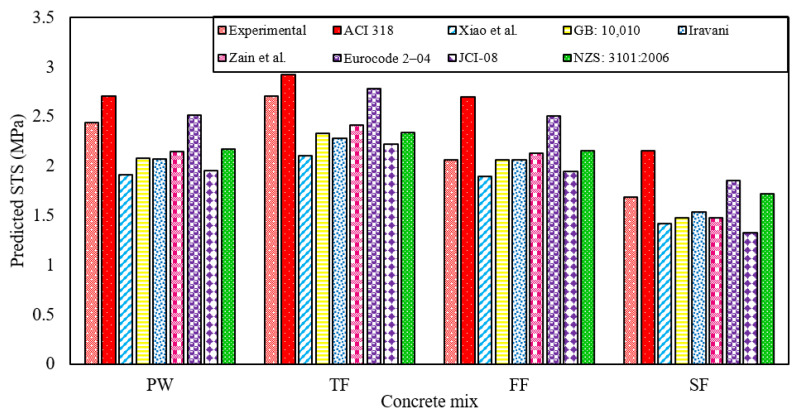
Comparison of predictions of various equations for the STS for RAGC mixes.

Figure 6 reports the experimental relationship between the CS and STS of various RAGC mixes. New mathematical models reporting the relationships between CS and STS of RAGC mixes made with different types of effluents were also presented in Figure 6. The predictions of various previously proposed models (reported in Table 5) describing the relationships between the CS and STS of plain concrete mixes at the testing ages of 28-days were compared with the experimental results of various RAGC mixes. The comparative study between the experimental results and the predictions of various models for predicting the STS of RAGC mixes was reported in Figure 7.

The model given by ACI 318 [52] portrayed the % age discrepancies of 11.25%, 7.94%, 30.85%, and 27.91% for FW, TF, FF, and SF mixes, respectively. These deviations were the highest as compared with the predictions of the other models. The highest discrepancy for ACI 318 [52] may be ascribed to the reason this model was recommended for the normal strength concrete made with freshwater having NCA but, in the present research, RCA was employed for the fabrication of RAGC mixes. The Xiao et al. [53] model portrayed the % age discrepancies of 21.65%, 22.25%, 7.96%, and 15.89% for FW, TF, FF, and SF mixes, respectively. The model proposed by GB: 10,010 [54] represented the % age discrepancies of 14.65%, 14%, 0.19%, and 12.47% for FW, TF, FF, and SF mixes, respectively. The Iravani [55] model portrayed the % age discrepancies of 15%, 15.65%, 0.15%, and 8.76% for FW, TF, FF, and SF mixes, respectively. The Zain et al. [56] model depicted the % age discrepancies of 11.89%, 10.95%, 3.4%, and 12.5% for FW, TF, FF, and SF mixes, respectively. Similarly, the model suggested by Eurocode 2-04 [57] showed the % age discrepancies of 3.3%, 2.77%, 21.33%, and 10% for FW, TF, FF, and SF mixes, respectively. The model suggested by JCI-08 [58] displayed the % age discrepancies of 19.64%, 17.82%, 5.75%, and 21.27% for FW, TF, FF, and SF mixes, respectively. And lastly, the anticipated model by NZS: 3101:2006 [59] demonstrated the % age discrepancies of 11%, 13.66%, 4.68%, and 2.33% for FW, TF, FF, and SF mixes, respectively. Therefore, the present study, after the proposed models in the present study that presented the highest accuracies, suggests using Eurocode 2-04 [57] for predicting the STS of FW mix and TF mix, JCI-08 [58] for predicting the STS of FF mix, and NZS: 3101:2006 [59] for predicting the STS of SF mix.

### 3.4. Chloride Ion Migration (CIM)

It reflects the degree of penetration into the concrete by chloride ions. Seawater chloride and de-icing salts enter the concrete, thereby affecting its functionality and performance, resulting in the deterioration of reinforced concrete buildings [60]. For this reason, CIM is a critical parameter for concrete strength. As the content of chloride ions on the surface of reinforcing steel goes up, corrosion begins as well as oxide formation happens, leading to changes in volume (due to the formation of iron rust) and this contributes to the spalling of the concrete cover. During process-oxidation of Fe in effluent chloride ion (${\mathrm{Cl}}^{-}$) serves as a catalyst forming ${\mathrm{FeCl}}_{3}^{-}$ complex. This complex being unstable forms iron hydroxide $\mathrm{Fe}{\left(\mathrm{OH}\right)}_{2}$ after reacting with the hydroxide ions (${\mathrm{OH}}^{-}$). After reacting with the hydroxide ions (${\mathrm{OH}}^{-}$), this volatile complex form iron hydroxide $\mathrm{Fe}{\left(\mathrm{OH}\right)}_{2}$. As a cause, the chloride ion turns up to the solution for the intake of hydroxyl ions free of charge. The iron hydroxide $\mathrm{Fe}{\left(\mathrm{OH}\right)}_{2}$ lowers the pH value that causes the oxide film to decrease and encourages the chloride ions (${\mathrm{Cl}}^{-}$) to penetrate freely. The following reactions (Equations (1)–(4))occur in concrete susceptible to CIM during the corrosion of steel bars.

(1) $$Fe\to Fe^{2+}+2e^{-}$$

(2) $$2H_{2}O+O_{2}+4e^{-}\to 4OH^{-}$$

(3) $$2Fe+6Cl^{-}\to 2FeCl_{3}^{-}+4e^{-}$$

(4) $$FeCl_{3}^{-}+2OH^{-}\to Fe{\left(OH\right)}_{2}+3Cl^{-}$$

**Figure 8:**
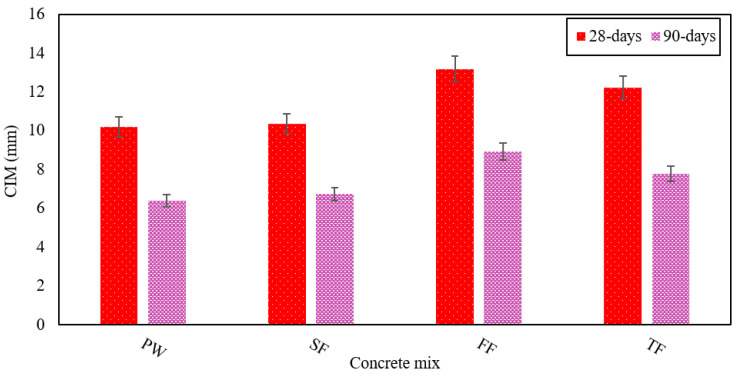
CIM in all RAGC mixes at 28 and 90 days of testing.

In this study, CIM to concrete is analyzed using 4% NaCl. The standard used for calculating this parameter is the depth in millimeters of the concrete matrix that such chloride ions invade. Figure 8 clearly shows the penetration values of chloride ions for all various RAGC mixes. The highest CIM values were provided by the FE, which is rich in chloride ion and sulfate ions.

The control mix had shown a CIM of 10.2 mm at 28 days and 6.4 mm at 90 days. The TF mix demonstrated CIM that was 16% and 17% superior to that of FW after 28 and 90 days, separately. This proves that the TF blend is more vulnerable to the oxidation of steel bars and rust. The CIM shown by the FF mix was 13.2 mm for 28 days and 9 mm for 90 days, separately. Due to rich in chloride and sulfate, the FE induces corrosion and faster penetration of chloride ions into concrete [61]. The chloride ion penetration is also risen due to the very low pH of the FE mix [51]. Thus, the chloride ion penetration shown by SE was the least among all the types of effluent examined, which indicates that it is less corrosion-resistant, whereas the FE showed the highest values for penetration of chloride ion and is, therefore, more prone to corrosion. The fly ash results in a decrease in the CIM due to the development of CSH-gel and the filling of voids between the cement and sand particles giving a denser matrix that prevents the CIM from the RAGC mix.

### 3.5. Resistance against Acid Attack

**Figure 9:**
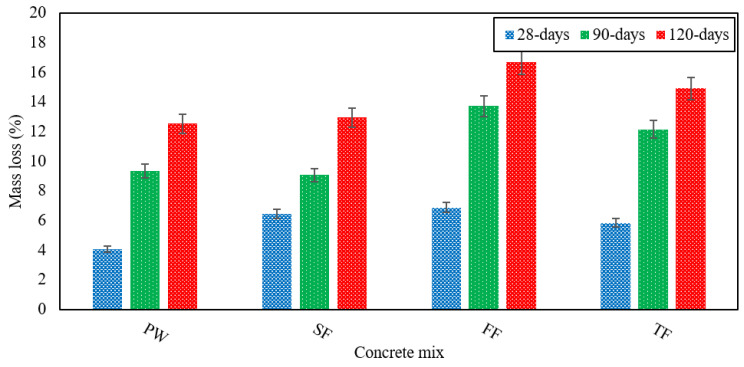
Mass loss of various RAGC mixes due to the attack of H_2_SO_4_ at 28, 90, and 120 days of testing.

It is very susceptible to acid attack because of the alkaline nature of concrete. As these acid attacks occur in the drainage system, they cause effluent to deteriorate. That is why the tolerance to acid attacks becomes an essential parameter of durability that must be observed. H_2_SO_4_ is the most violent and damaging acid among all the other acids and having a low pH. It easily reacts with CH forming CaSO_4_ leads to rapid degradation of concrete. This study examined the mass loss of specimens taken as concrete degradation at 28, 90, and 120 days. Later, they were submerged in a 4% H_2_SO_4_ solution. In Figure 9, the mass loss experienced by each of the RAGC mixes is presented. For the FF mix, the highest degradation was observed.

**Figure 10:**
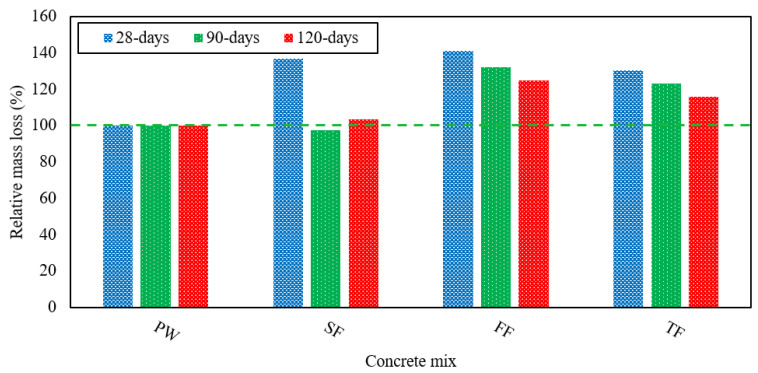
Mass loss of various RAGC mixes relative to FW at 28, 90, and 120 days of testing.

The TF mix shows faster deterioration associated with the design control mix. At 28 days, the TF revealed mass losses of 5.8%, 12% at 90 days, and 15% at 120 days, which were on average 30%, 23%, and 16% better than to the control mix. The FF mix recorded mass losses of 6.8% at 28 days, 13.7% at 90 days, and 16.7% at 120 days, which were on average 40%, 32%, and 24% superior to the control mix. Concrete deterioration is also mainly influenced due to the pH value of acid and the mixing of effluent. The significant mass loss could be due to the sulfate-rich in FE. The FF mix exhibited the highest deterioration in the initial days, but the deterioration was very close to that of FW at 90 and 120 days. Due to the H_2_SO_4_ attack, the SF mix showed higher mass losses that were 6.4% at 28 days, 9% at 90 days, and 13% at 120 days separately. Thus, we can conclude that with the use of all the various forms of effluent that were studied, the degradation of concrete becomes more severe. The relative mass losses of different concrete mixes due to acid attack at several testing ages are shown in Figure 10. The adding of fly ash in the GPC fills the micro-cracks and results in a decrease in the acid absorption of RAGC mixes because of filler and the development of CSH-gel after the pozzolanic reaction of CH and fly ash particles. Consequently, the penetration of H_2_SO_4_ in RAGC will be less giving less loss of mass at various testing ages.

### 3.6. Relationship Analysis of Parameters

**Figure 11:**
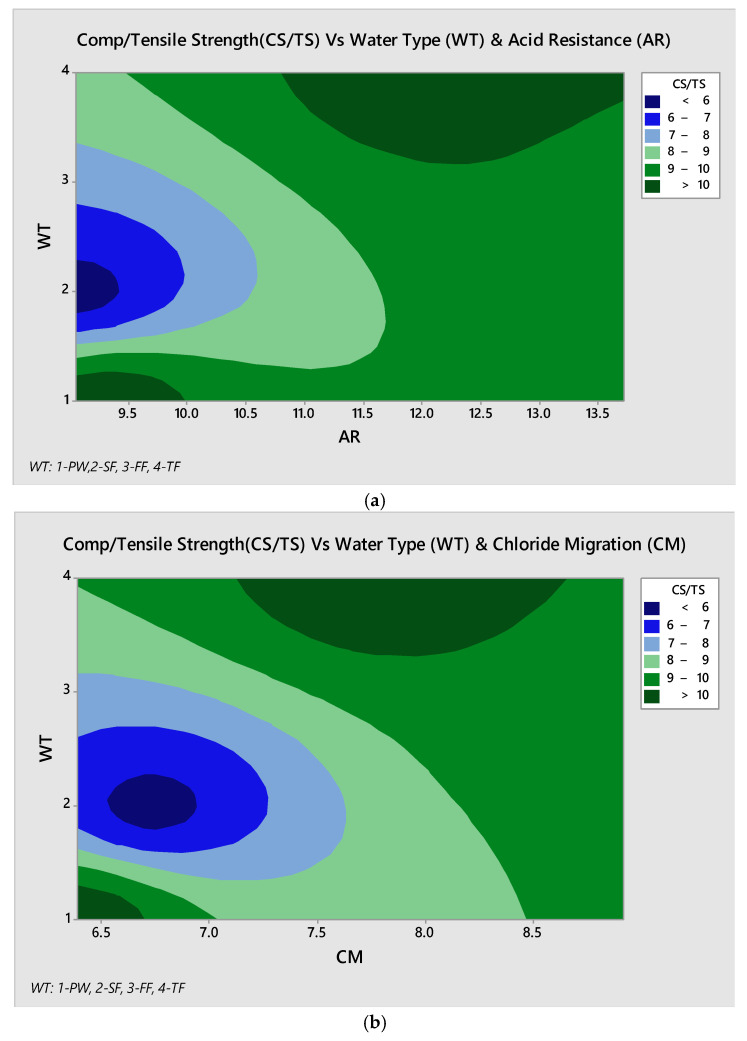
Relationship of CS/TS vs (**a**) acid resistance & (**b**) chloride migration at 90 days.

It is necessary to study the relationship pattern of strength parameters with reference to water type utilized and performance analysis of developed concrete under different environmental conditions like acid resistance and chloride migration. These parameters are linked with natural soil and environmental conditions. So, the compressive to tensile strength ratio has been studied with reference to water type utilization and Acid resistance. From Figure 11, it can be observed that at 90 days of strength conditions, textile wastewater produces higher strength and better resistance to maintain its strength level. Similarly, under chloride migration condition there is a greater trend of chloride migration concerning water type and especially textile water produces higher strength level under varying circumstances. However, sugar mill wastewater has performed in lower-order by performing towards lower strength development against both acid resistance and chloride migration conditions.

### 3.7. Financial Feasibility Analysis of Concrete

During the development of sustainable concrete, the focus is to produce concrete using waste products and recycled aggregates, but some ingredients used as a replacement of cement can also contribute to low-cost concrete with a suitable strength as shown in Table 6. Hence, the price of plain normal strength concrete is 13.78% higher than the price of recycled aggregate geopolymer concrete.

### 3.8. Statistical Test Results

In this study, the Analysis of Variance was used to evaluate the significance of the differences between the different durability characteristics of the RAGC mixes at 90 days as well as mechanical properties at 28 days testing, as presented in Table 7, Table 8, Table 9 and Table 10. Table 7 reports the ANOVA analysis of CS for various RAGC mixes. Table 7 reports the ANOVA test for the STS of various RAGC mixes. Table 8 portrays the ANOVA predictions for the CIM and Table 9 presents the ANOVA analysis results for the mass loss of various RAGC mixes. Three samples were collected from each group, the RAGC mix was divided into four groups such as FW, TF, FF, and SF. A contrast between all the RAGC mixes and the control mix (FW) was produced to present the significance of the experimental outputs accurately. A *p*-value of more than 5% in the ANOVA test at a confidence interval of 95% and less F than $F_{crit}$ indicates that the discrepancy between the outcomes of the various RAGC mixes is negligible. In this case, the results of the ANOVA test at a sign. level of 5% showed that the various RAGC mixes showed a substantial difference (p=0.045% and $F>F_{crit}$) between their CS at 28 days of testing, indicating that the CS of RAGC was greatly affected by studied effluent forms. Although on the other hand, the findings of the split tensile test (p=19.63% and $F<F_{crit}$), and the sulphuric acid attack test (p=6.77% and $F<F_{crit}$) showed no substantial difference between such RAGC mixes. The results of the CIM of different RAGC mixes have shown no substantial difference between them with a probability of 8.84% and $F<F_{crit}$ which indicates that the forms of effluent studied in RAGC mixing influenced the results of the acid attack.

## 4. Conclusions

The current study aimed to study and analyze the performance properties of recycled aggregate geopolymer concrete generated using several types of effluent (sugar mill effluent, manure mill effluent, and textile mill effluent). This study investigated the effects of 100% replacement of all these various forms of effluent on CS, STS, and CIM, and sulfuric acid attack on recycled aggregate geopolymer concrete produced from freshwater. A one-way ANOVA test was conducted out to investigate the implication of discrepancies between the properties of different recycled concrete aggregate mixes. The experimental results give the following important conclusions:

1. The CS demonstrated by the RAGC mix produced using textile mill effluent was 25% higher on average than the CS of concrete created with freshwater. Due to the presence of mixed organic waste that seems to absorb water, the CS decreased to about half of its value when sugar mill effluent has been used. The RAGC mixes produced using FE gave the highest CS of 91% and the strength shown by the RAGC mix of SE was 90% relative to that revealed by RAGC produced by freshwater. The formation of CSH gel in the RAGC gave the improved CS of mixes.
2. Tests conducted to analyze STS found that concrete produced using textile mill effluent showed higher strength (about 17% higher) than the tensile strength of concrete produced using freshwater. The RAGC mixes produced using effluent from fertilizer mill exhibited the best STS of 97%, the mix produced by sugar mill effluent gave a maximum tensile strength of 92% while the strength shown by the RAGC mix of SE was 95% compared to concrete produced with freshwater.
3. The testing results of CIM found that the highest level of CIM was shown by concrete produced using effluent of fertilizer mill, i.e., about 13.2 mm at 28 days, while 9 mm at 90 days especially in comparison to concrete produced with freshwater. The RAGC blends made using effluent of textile mill at 120 days gave the highest CIM of 117%, the mix of sugar mill effluent had the highest CIM of 102% while the CIM shown by the RAGC mix of SE was 108% when compared with concrete produced using freshwater. The micro-structure of RAGC did not allow chloride ions to penetrate in the concrete matrix due to the high density of GPC.
4. The results of tests conducted by exposing RAGC mixes to 4% H_2_SO_4_ solution revealed that the uppermost mass loss with reference to acid attack was seen in the RAGC mix created using effluent from the fertilizer mill, such as 17% at 120 days, and this could be understood by the fact that the pH value of effluent from the fertilizer mill was the lowest because a decrease in pH value increases the mass loss of concrete. The RAGC mixes produced using textile mill effluent at 120 days displayed the highest mass loss of 116%, the mix produced by sugar mill effluent gave the highest mass loss of 103% while the mass loss shown by the RAGC mix of SE was 112% separately as compared to that concrete made with freshwater. The RAGC mixes showed improved results for the resistance against acid attack due to the dense matrix provided by the fine particle of GPC.
5. The ANOVA test illustrated major variations in the effects of different RAGC mixes for CS. Likewise, there were no such major variations in the STS, CIM, and acid attack resistance of RAGC mixes. To conclude, the forms of effluent that have been studied can be used to build sustainable concrete in terms of the selection of waste materials and a green environmental impact.
6. Therefore, the RAC with waste effluents can be used for the production of green concrete (GPC) to be used for the large-scale concrete construction in various structural elements portraying significant mechanical and durability properties.

## 5. Limitations of the Study

In this study different types of effluents have been used to develop recycled aggregate geopolymer concrete which is an environment-friendly solution to develop suitable construction materials. With the variation of chemical formation of effluents, the performance of developed concrete may vary so repetitive testing regarding physio-chemical properties of effluent can be done to find matching effluents as replacement of water.

## Figures and Tables

**Table 1:** Different properties of recycled aggregates.

Parameter	Value	Parameter	Value
Bulk density	1302 kg/m^3^	Los Angeles abrasion	38.54%
10% fine value	142	Minimum size	4.75 mm
Apparent density	1723 kg/m^3^	Specific gravity	2.25
WA at 24 h	6.62%	Maximum size	10 mm

**Table 2:** Characteristics of fly ash.

Chemical		Physical	
Compound	Quantity (%)	Parameter	Value
SiO_2_	55.4	Consistency [42]	29.2%
Fe_2_O_3_	3.3	Fineness (Blaine Test)	2767 (cm^2^/g)
Na_2_O	1.8	Soundness [43]	No expansion
CaO	3.9	Specific surface area [44]	387 m^2^/kg
Al_2_O_3_	29.8	–	–
MgO	1.6	–	–
SO_3_	1.4	–	–

**Table 3:** Physical and chemical properties of all types of effluent studied in the present work.

Parameter (Unit)	FW	SE	FE	TE
pH value	7.0	7.2	2.5	7.0
TSS (mg/L)	27.9	459	50.4	19.8
TDS (mg/L)	806.4	986.4	4699.8	344.7
COD (mg/L)	16.8	378.9	940.5	108
DO (mg/L)	5.7	2.6	2.2	4.8
BOD (mg/L)	11.1	279.9	549	63
Hardness (mg/L)	325.8	648.9	2304	307.8

**Table 4:** Mix design of GRAGC (kg/m^3^).

Material	Quantity	Material	Quantity
RCA	1107	Fly ash	247
Water	126	Superplasticizer	39
Sand	499	GGBS	165
NaOH solution (14M)	41	Na_2_SiO_3_	107

**Table 5:** Relationships between CS and STS of plain concrete available in the literature.

Standard/Research	Formula
ACI 318 [52]	$f_{sts}=0.55\times \sqrt{f_{c}^{\prime }}$ (MPa)
Xiao et al. [53]	$f_{sts}=0.24\times f_{c}^{\prime }{}^{0.65}$ (MPa)
GB: 10,010 [54]	$f_{sts}=0.19\times f_{c}^{\prime }{}^{0.75}$ (MPa)
Iravani [55]	$f_{sts}=0.301\times {\left(0.8f_{c}^{\prime }\right)}^{0.65}$ (MPa)
Zain et al. [56]	$f_{sts}=\frac{0.8f_{c}^{\prime }}{0.1\times \left(0.8f_{c}^{\prime }\right)+7.11}$ (MPa)
Eurocode 2–04 [57]	fsts=0.30×fc′23 (MPa)
JCI-08 [58]	$f_{sts}=0.13\times f_{c}^{\prime }{}^{0.85}$ (MPa)
NZS: 3101:2006 [59]	$f_{sts}=0.44\times \sqrt{f_{c}^{\prime }}$ (MPa)

**Table 6:** Financial Comparative Analysis of PNSC and RAGC for 1 m Cube of Concrete.

Plain Normal Strength Concrete (1 m^3^)				Recycled Aggregate Geopolymer Concrete (1 m^3^)			
Ingredients	Unit Price (USD)	Unit Weight (kg)	Price (USD)	Ingredients	Unit Price (USD)	Unit Weight (kg)	Price (USD)
	A	B	A × B		A	B	A × B
NCA	0.04	1188	52	RCA	0.04	1107	48
Water	0.12	165	20	Water	0.12	126	16
Sand	0.03	637	20	Sand	0.03	499	15
Superplasticizer	0.06	3.9	0	Superplasticizer	0.06	3.9	0
Cement	0.07	451	34	Fly ash	0.04	247	11
–	–	–	–	GGBS	0.03	165	5
–	–	–	–	NaOH solution	0.12	41	5
–	–	–	–	Na_2_SiO_3_	0.07	107	8
Total price=		USD	126	Total price=		USD	108

**Table 7:** ANOVA for the CS of RAGC mixes made with different types of effluent at 28-days.

Groups.	Count.	Sum.	Average.	Variance.		
FW	3	72.9795	24.3265	2.97598744		
TF	3	96.822	32.274	6.28107975		
FF	3	71.38725	23.79575	5.13139069		
SF	3	49.45875	16.48625	10.5966053		
ANOVA						
SOV	SS.	df.	MS.	F.	*p*-value.	$F_{\mathrm{crit}}$
Between. Groups.	374.6074014	3	124.8691338	19.9910055	0.00045	4.066181
Within Groups.	49.97012625	8	6.246265781			
Total	424.5775277	11				

**Table 8:** Results of ANOVA test for the STS of RAGC mixes made with different types of effluent at 28-days.

Groups.	Count.	Sum.	Average.	Variance.		
FW	3	7.013	2.3375	0.001383937		
TF	3	8.225	2.74175	0.236676		
FF	3	6.757	2.25225	0.070036313		
SF	3	6.567	2.189	0.067540688		
ANOVA						
SOV	SS.	df	MS.	F	*p*-value	$F_{\mathrm{crit}}$
Between Groups.	0.556411	3	0.185470313	1.97499547	0.196382109	4.066180551
Within Groups.	0.751274	8	0.093909234			
Total	1.307685	11				

**Table 9:** Results of ANOVA test for CIM of RAGC mixes made with different types of effluent at 90-days.

Groups.	Count.	Sum.	Average.	Variance.		
FW	3	19.1604	6.3868	0.77053872		
TF	3	23.3268	7.7756	3.20768112		
FF	3	26.7624	8.9208	0.54980352		
SF	3	20.1768	6.7256	0.51327696		
ANOVA						
SOV	SS.	df	MS.	F.	*p*-value.	$F_{\mathrm{crit}}$
Between Groups.	11.77319	3	3.92439824	3.113798418	0.0884	4.066180551
Within Groups.	10.0826	8	1.26032508			
Total	21.8558	11				

**Table 10:** Results of the ANOVA test for the sulfuric acid attack of RAGC mixes made with diff. types of effluent at 90-days.

Groups.	Count.	Sum.	Average.	Variance.		
FW	3	37.565325	12.521775	0.4132		
TF	3	44.741025	14.913675	0.4594		
FF	3	50.042475	16.680825	11.138		
SF	3	38.8416	12.9472	0.4481		
ANOVA						
SOV	SS.	df	MS.	F.	*p*-value.	$F_{\mathrm{crit}}$
Between Groups.	33.09725	3	11.03241691	3.5421	0.067756953	4.066180551
Within Groups.	24.91745	8	3.114680973			
Total	58.0147	11				

## Abbreviations

CS = compressive strength;
STS = split tensile strength;
RAGC = recycled aggregate geopolymer concrete;
SE = sugar mill effluent;
FE = fertilizer mill effluent;
TE = textile mill effluent;
CIM = chloride ion migration;
GPC = geopolymer concrete;
RAC = recycled aggregate concrete;
RCA = recycled coarse aggregate;
FW = freshwater mix;
SF = sugar mill effluent mix;
FF = fertilizer mill effluent mix;
TF = textile mill effluent mix.
